# Correction: Machine learning-based e-commerce platform repurchase customer prediction model

**DOI:** 10.1371/journal.pone.0315518

**Published:** 2024-12-05

**Authors:** Cheng-Ju Liu, Tien-Shou Huang, Ping-Tsan Ho, Jui-Chan Huang, Ching-Tang Hsieh

There are errors in the author affiliations. The correct affiliations are as follows:

Cheng-Ju Liu^1^, Tien-Shou Huang^1^, Ping-Tsan Ho^2^, Jui-Chan Huang^3^, ChingTang Hsieh^4^

**1** Department of Intelligent Commerce, National Kaohsiung University of Science and Technology, Kaohsiung City, Taiwan, **2** Department of Leisure and Sport Management, College of Life and Creativity, Cheng Shiu University, Kaohsiung City, Taiwan, **3** Yango University, Fuzhou, China **4** Department of International Business, National Kaohsiung University of Science and Technology, Kaohsiung City, Taiwan
